# Design and Fabrication of a Biomimetic Smart Material for Electrochemical Detection of Carbendazim Pesticides in Real Samples with Enhanced Selectivity

**DOI:** 10.3390/bios14060304

**Published:** 2024-06-10

**Authors:** Francisco Franciné Maia Júnior, Rui Sales Junior, Geovani Ferreira Barbosa, Sajjad Hussain, Eduardo Jara-Cornejo, Sabir Khan

**Affiliations:** 1 Department of Natural Sciences, Mathematics, and Statistics, Federal Rural University of the Semi-Arid, Mossoró 59625-900, RN, Brazil; 2 Faculty of Materials and Chemical Engineering, GIK Institute of Engineering Sciences and Technology, Swabi 23640, Pakistan; sajjad.hussain@giki.edu.pk; 3 Technology of Materials for Environmental Remediation (TecMARA) Research Group, Faculty of Sciences, National University of Engineering, Av. Tupac Amaru 210, Rimac, Lima 15032, Peru; ejjarac@uni.pe; 4 Department of Exact Sciences and Technology, State University of Santa Cruz, Ilhéus 45662-900, BA, Brazil

**Keywords:** molecularly imprinted polymers (MIP), carbendazim, biomimetic sensor, HPLC

## Abstract

Agricultural products are vitally important for sustaining life on earth and their production has notably grown over the years worldwide in general and in Brazil particularly. Elevating agricultural practices consequently leads to a proportionate increase in the usage of pesticides that are crucially important for enhanced crop yield and protection. These compounds have been employed excessively in alarming concentrations, causing the contamination of soil, water, and air. Additionally, they pose serious threats to human health. The current study introduces an innovative tool for producing appropriate materials coupled with an electrochemical sensor designed to measure carbendazim levels. The sensor is developed using a molecularly imprinted polymer (MIP) mounted on a glassy carbon electrode. This electrode is equipped with multi-walled carbon nanotubes (MWCNTs) for improved performance. The combined system demonstrates promising potential for accurately quantifying carbendazim. The morphological characteristics of the synthesized materials were investigated using field emission scanning electron microscopy (FESEM) and the Fourier-transform infrared (FTIR) technique. The analytical curve was drawn using the electrochemical method in the range of 2 to 20 ppm while for HPLC 2–12 ppm; the results are presented as the maximum adsorption capacity of the MIP (82.4%) when compared with NIP (41%) using the HPLC method. The analysis conducted using differential pulse voltammetry (DPV) yielded a limit of detection (LOD) of 1.0 ppm and a repeatability of 5.08% (*n* = 10). The results obtained from the analysis of selectivity demonstrated that the proposed electrochemical sensor is remarkably efficient for the quantitative assessment of carbendazim, even in the presence of another interferent. The sensor was successfully tested for river water samples for carbendazim detection, and recovery rates ranging from 94 to 101% were obtained for HPLC and 94 to 104% for the electrochemical method. The results obtained show that the proposed electrochemical technique is viable for the application and quantitative determination of carbendazim in any medium.

## 1. Introduction

By 2009, the global gain of using pesticides already crossed the mark of USD 37.86 billion, out of which USD 7.7 billion was attributed to sales in Latin America, while Brazil was accountable for 85% of this demand. It is crucial to highlight that pesticide consumption can be categorized into various types, for instance, insecticides, herbicides, fungicides, etc. Fungicides find applications in agriculture to manage plant diseases caused by fungi, which can substantially diminish both crop yields and quality [1,2]. Pesticides are categorized based on the target pest. They are subdivided into insecticides, acaricides, nematicides, rodenticides, fungicides, and herbicides [3,4,5].

Carbendazim (CBZ) is a fungicide belonging to the benzimidazole family that is incorporated in both pre- and post-harvest stages to inhibit the spread of fungal diseases in a variety of crops [6,7]. CBZ is able to distribute placidly and evenly from the site of application into the groundwater system, where it can eventually cause severe environmental hazards to almost all types of ecosystems. They pose a potential threat to animals and aquatic life, even at very low concentrations. Pesticides have been reported to likely cause a wide range of health issues in humans, including teratogenic and carcinogenic effects, so it is inevitable that CBZ is detected at extremely low concentrations to guarantee public health and prevent environmental contamination [8]. This is because CBZ has a significant level of chemical stability because of the benzimidazole ring [7,9,10]. The identification of pesticide residues is crucial in order to forestall harmful problems brought about by the increased use of pesticides in agriculture [11].

**Figure 1:**
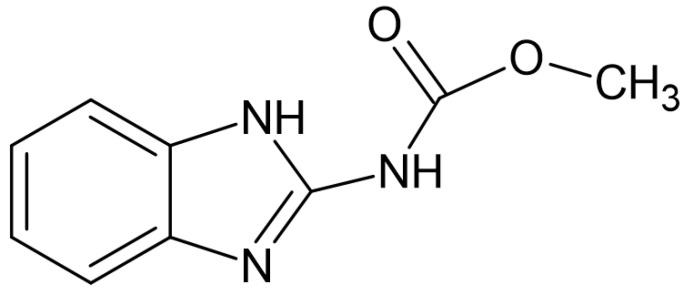
Representation of the carbendazim molecule.

It is important to highlight that the CCDC (Cambridge Crystallographic Data Centre) provides data exclusively for crystals. The atomic coordinates utilized for constructing the carbendazim molecule, as shown in Figure 1, were taken from the data associated with the carbendazim crystal that exists in the CCDC repository. 

The detection of CBZ has been investigated using a wide range of analytical methods including high-performance liquid chromatography (HPLC) [12,13], gas chromatography (GC) [5,9], liquid chromatography (LC) coupled with mass spectrometry (LC/MS–MS) [14,15], electrochemical techniques [16,17,18], and fluorescent sensors [19]. Additionally, various sample preparation procedures have been employed.

The electrochemical method offers distinctive features by virtue of its selectivity and high sensitivity when applied to the CBZ determination process. The safety of humans and ecosystems depends on the rapid development of selective and reliable trace-level extraction techniques for its residues in the wastewater matrix.

Molecularly imprinted polymers (MIPs), which are receptors crafted through a template-oriented manufacturing approach, possess unique cavities that are molded by the template itself. These cavities enable MIP to effectively rebind the target analyte, even within intricate matrices. These synthetic polymers are made through the copolymerization of a template and a functional monomer using a cross-linker [20,21,22]. In this regard, there has been a lot of interest in using MIP in conjunction with other materials as core–shell nanomaterials as a result of its synergistic impact that allows for a rapid exchange of mass. MIP cavities are more distinctively visible in these hybrid materials rather than in traditional MIPs [23].

MIPs are ideal for use as sorbents in the detection of trace levels of pesticides because of their many intrinsic benefits, including their high selectivity, sensitivity [24], reusability, physicochemical stability [25], and cost-effectiveness [26]. In recent years, there has been a dramatic increase in the fascination of utilizing molecularly imprinted polymers (mag-MIP) in environmental analysis. This material has been effectively employed for this purpose, facilitating a precise, reliable, and selective identification of a diverse and extensive spectral domain of analytes within real-world samples. Consequently, MIP is being regarded as an emerging candidate for monitoring CBZ.

This study details the development of the electrochemical sensor coupled with MIP/NIP that can be subjected to CBZ monitoring. Synthesized polymer materials were subsequently employed for the analysis of actual samples.

## 2. Materials and Methods

### 2.1. Chemicals and Reagents

All materials and reagents used were of HPLC or analytical grade. The Milli-Q Direct-0.3 purifier was used to obtain deionized water (18 M cm^−1^t 25 °C, Millipore, Burlington, MA, USA). Carbendazim, along with the following chemicals used in the synthesis of the molecularly imprinted polymer, were exclusively sourced from Sigma-Aldrich Brazil: ethanol, 1 vinyl imidazole, ethylene glycol dimethacrylate, 4,4′-Azobis-4-cyanopentanoic acid (ACPA), acetic acid, NaOH, and HCl. After preparing a carbendazim stock solution in 100 ppm working solutions with minor concentrations ranging from 2.0 to 12 ppm, the stock solution was diluted with deionized water (pH 7) in 10 mL volumetric flasks.

### 2.2. Instruments

Morphological analyses were carried out using an SEM (VEGA 3 LMU, Tescan, Brno-Kohoutovice, República Tcheca) scanning electron microscope.

An FTIR-Vertex 70 spectrophotometer (Bruker, Billerica, MA, USA) with a spectral range of 4000 to 400 cm^−1^ was used to analyze the functional groups of the synthesized products.

All the electrochemical analyses were conducted in a 10 mL volume electrochemical cell. The analyses were accomplished using the AutoLab potentiostat model (μAutolab type III, Autolab/Eco Chemie), and the NOVA 2.2 software was employed to control the voltammetric measurements. The electrochemical cell involved the assembly of the following components: a reference electrode (Ag/AgCl/KCl sat), an auxiliary electrode made of platinum wire, and a working electrode utilizing carbon multi−walled carbon nanotubes (MWCNTs) and MIPs. The chromatographic analysis was carried out using an ultra-performance liquid chromatography (HPLC) model, Shimadzu 20A, coupled with the SPD-20A UV detector and a C18 column measuring 250 × 4.6 mm with a 5 μm particle size and 100 Å pore size (Kinetex from Phenomenex, Torrance, CA, USA). The mobile phase comprised 1% formic acid and acetonitrile (60:40, *v*/*v*); at a flow rate of 0.8 mL min^−1^, the injection volume was 20 μL and the wavelength set was 260 nm. The retention time for carbendazim was approximately 2.5 min.

### 2.3. Synthesis of Molecularly Imprinted Polymer (MIP)

MIP was synthesized by polymerization with (0.2 mmol) CBZ as the analyte and (0.8 mmol) 1-vinyl imidazole as the functional monomer in 30 mL of ethanol, and then 30 mL of deionized water was added. The reaction mixture was stirred constantly at room temperature for 3 h to induce a complete reaction. After adding the crosslinking agents EGDMA (4 mmol) and the radical initiator, 4,4′-Azobis-4-cyanopentanoic acid (0.050 mmol) was added. The reaction mixture was maintained at 60 °C for 24 h in an atmosphere of pure nitrogen bubbling to ensure an inert environment and yield the formation of the desired products.

Upon the completion of the polymerization process up to absolute dryness, the material was transferred to the Soxhlet apparatus to wash the final product using a 9:1 (*v*/*v*) mixture of methanol and acetic acid over a period of 3 days. The solvent was regularly altered at fixed intervals of 12 h and was analyzed until no peaks of the analyte appeared. Additionally, any unreacted chemicals were also removed. The final polymer materials were then collected and dried in an oven at 60 °C. The non-imprinted polymer was synthesized following the same protocol without the use of CBZ [22].

### 2.4. Modification of GCE with MWCNT and MIP

In order to enhance the sensor’s analytical signal response, a solution containing 1 mg of MWCNT was mixed with 1 mg each of MIP and NIP in 1 mL of water. The mixture was subjected to sonication in an ultrasound bath for 30 min, and then 5 µL of nafion was added. Finally, 5 µL of the resulting dispersion was deposited onto the electrode surface. An IR lamp was employed to thoroughly evaporate the solvent up to dryness and act as a working electrode.

### 2.5. Adsorption Process

Binding experiments were performed using 10.0 mL vials containing 4.0 mg of the MIP (or NIP) and 5.0 mL of CBZ at final concentrations of (2, 4, 6, 8, 10, and 12 ppm). The parameters, including mass, concentration, and contact time, were optimized. After shaking for 90 min on a rotating shaker, the polymer materials were separated from the mixtures using Whatman filter paper. High-performance liquid chromatography with UV detection (HPLC-UV) at 260 nm was employed, utilizing a mixture of formic acid and acetonitrile for analysis.

## 3. Results

### 3.1. Morphology of MIP

Scanning electron microscopy (SEM) was used to assess the variations in the surface morphology of the MIP. The MIP and NIP particles were mounted on a metal sample holder using carbon conductive tape and sputtered with a 9 nm thick gold layer. This procedure is used to enhance material conductivity, generating a higher-quality image. Images were captured with a secondary electron detector operating with an electron beam of 20 kV.

**Figure 2:**
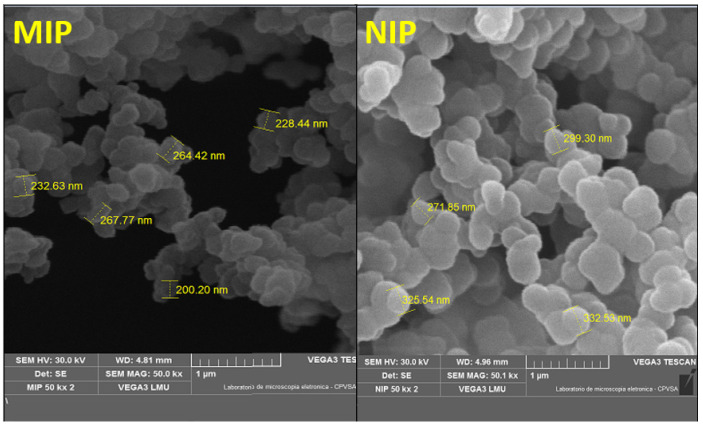
SEM image of MIP (left) and NIP (right).

Figure 2 illustrates the structure of the modified MIP, as observed through scanning electron microscopy. A more spherical shape was obtained for the MIP after a controlled synthetic protocol, and the material was found to be evenly dispersed throughout the nanomaterials’ surfaces. The surface of the molecularly imprinted polymer (MIP) exhibited enhanced binding sites and a distinctive structure designed for the specific recognition of the target compound when compared with NIP. Moreover, the MIP surface possessed a notably rough texture, resulting in a larger surface area and increased porosity. Molecularly imprinted materials possessed specific recognition sites that facilitated the effective capture of the target molecules.

The NIP is depicted in Figure 2; this control polymer material is identical in composition to the MIP, excluding the analyte. Figure 2 shows that the NIP profile bears a close resemblance to the MIP.

Through amplification of 50.0 kx times, it was concluded that NIP particles were slightly larger in size compared to the MIP particles. The average size of the imprinted polymer was 260.00 nm, which is almost similar to those of the non-imprinted polymer. It is evident from these images of MIP that it encompasses numerous effective imprinting sites, which facilitates the rebinding study as well as the selectivity of the material. The obtained structures and morphologies depicted the successful synthesis of the desired MIP.

**Figure 3:**
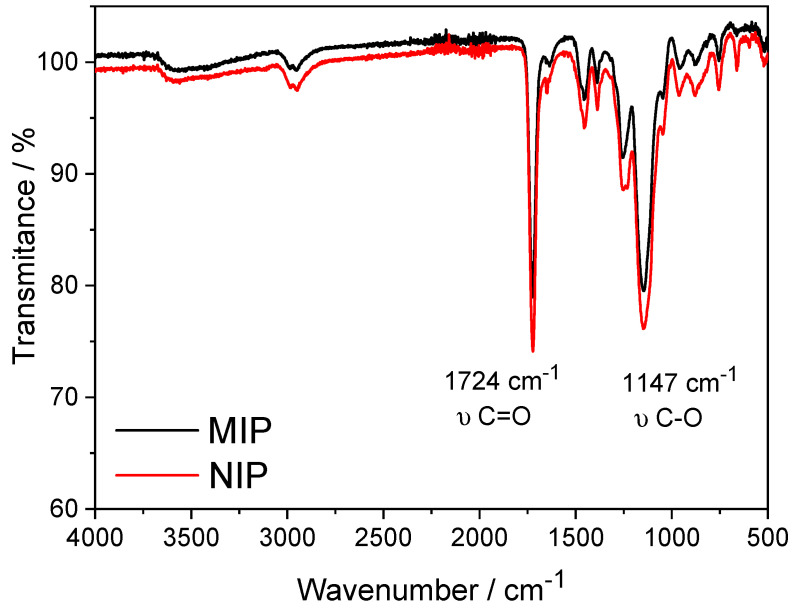
FTIR spectra of MIP (black) and NIP (red) produced in the presence of the 1− vinyl imidazole functional monomer.

On the other hand, the characteristic peaks in the infrared spectra of 1-vinyl imidazole are depicted in Figure 3. 1-vinyl imidazole reveals peaks at 3113 and 1548 cm^−1^, which can be attributed, respectively, to the (C-H) and (C=C) stretching vibrations in the vinyl group (-CH=CH2). The Ester group peaks at around 1724 cm^−1^ are associated with carbonyl (C=O) stretching since the MIP structure is mainly composed of EGDMA structural monomers, and the peak at 1147 cm^−1^ corresponds to the C-O bond of this monomer.

### 3.2. Construction of HPLC Curve

**Figure 4:**
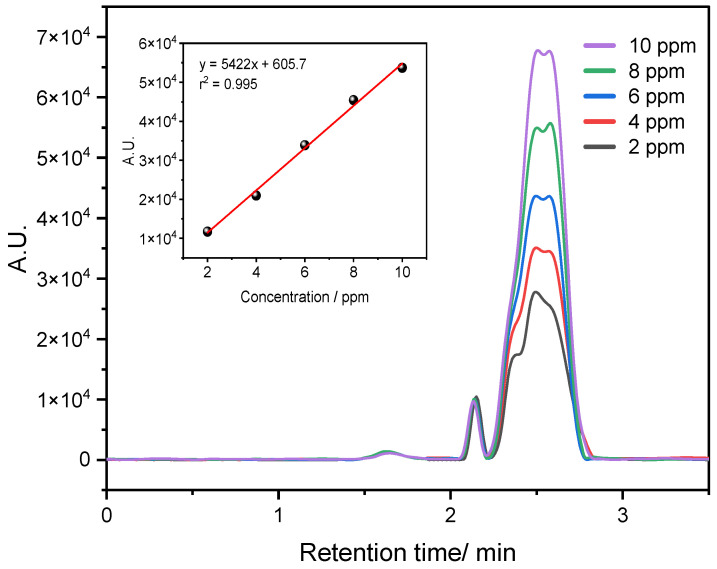
Chromatography profile of CBZ at a detection wavelength of 260 nm with a concentration range of 2.0 to 10.0 ppm. Insert: Calibration curve by HPLC method.

Figure 4 illustrates the analytical curve of the CBZ pesticide. The spectra were collected at the optimized condition of 260. An increase in pesticide concentration is accompanied by a corresponding rise in intensity. The concentration range produced a linear plot with a coefficient of determination (R^2^) of 0.994, slope = 5422 AU/ppm, and intercept = 605.7 AU.

### 3.3. Optimization of Concentration

**Figure 5:**
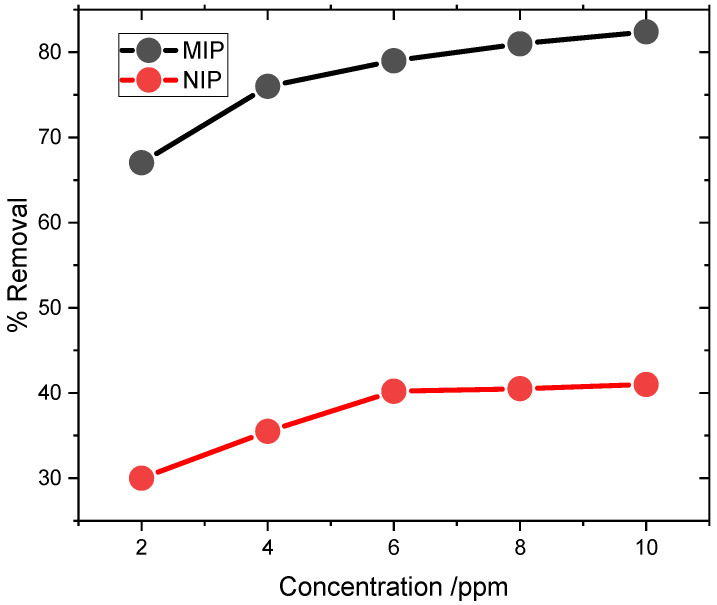
Optimization profile between MIP and NIP adsorption comparison. (Quantification technique was HPLC with a concentration range of 2.0 to 12.00 ppm).

The adsorption of carbendazim was evaluated through the application of MIP and NIP using 4 mg of the material mass at pH 7 for 90 min of continuous shaking. As can be seen in Figure 5, an increase in the concentration from 2 to 10 ppm resulted in a proportionate increase in percent adsorption. However, when a 10 ppm dose of MIP was applied, the surface of the adsorbent became almost saturated, and hence, no prominent percentage (%) removal could be observed for both MIP and NIP.

In addition, it is worth mentioning here that the percentage of the maximum adsorption capacity is relatively low, specifically at a value of 41.00% for NIP and 82.4% for MIP. This may be solely attributed to the absence of a template molecule in the synthesis of NIP and the lack of an imprinting site on their surface for the adsorption of CBZ on NIP.

### 3.4. Electrochemical Behavior of Modified Electrode

**Figure 6:**
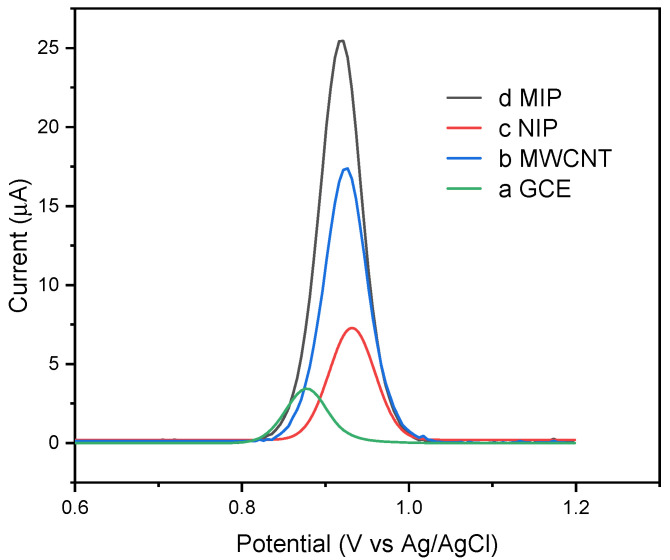
Differential pulse voltammograms obtained for (a) GCE, (b) MWCNT, (c) NIP/MWCNT, and (d) MIP/MWCNT in acetate buffer with acetonitrile (at pH 5) in the presence of 20 ppm carbendazim.

The electrochemical profiles were obtained for CBZ using differential pulse voltammetry in 0.1 mol L^−1^ of acetate buffer electrolytic solution (pH 5.0) with 10% acetonitrile, at a scan rate (υ) of 50 mV s^−1^, are shown in Figure 6. In all cases, there was an appearance of an oxidation peak during the anodic potential scanning and no reduction peak in the cathodic potential scanning. A better electrochemical signal was obtained for MIP/MWCNT compared to NIP/MWCNT and GCE. The signal was also tested with the carbon nanotube. This increase in the anodic current was due to the preconcentration of carbendazim on the MIP’s surface with selective capture of the analyte in the cavities of the MIP. Analogous responses for the three electrodes were obtained via differential pulse voltammetry. Similarly, an appropriate response was observed for NIP, but it was weaker compared to the well-dominant peaks of the modified MIP.

**Figure 7:**
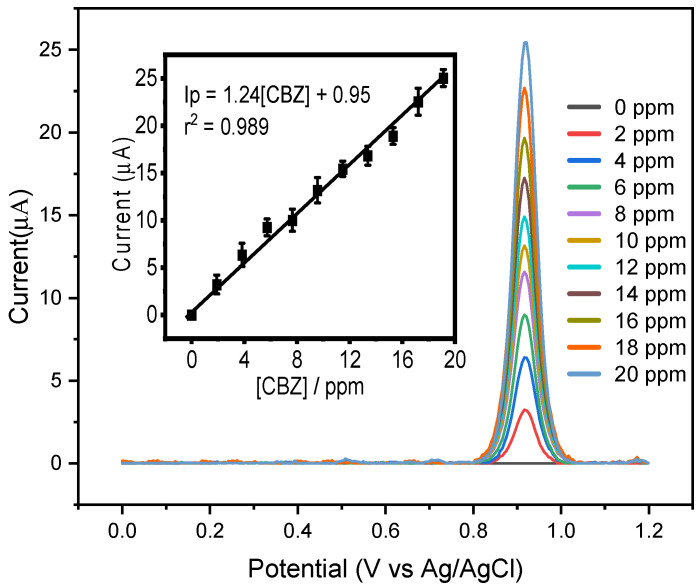
Differential pulse voltammogram graph obtained using the MIP/MWCNT sensor in 0.1 mol L^−1^ acetate buffer with acetonitrile (pH 5.0) containing different concentrations of the analyte ranging from 0 to 20 ppm (replicated *n* = 3). Inset: Electrochemical analytical curve.

Analytical voltammograms were recorded using all the optimized parameters during differential pulse voltammetry (DPV). In Figure 7, the corresponding voltammograms obtained from applying the proposed sensor modified with MIP and carbon nanotubes are depicted. Under optimized conditions, the sensor demonstrated a linear concentration range from 0 to 20 ppm with R^2^ equal to 0.989; to determine the detection limit, the value of the standard deviation (s) of the blank was first calculated with a quantity of *n* = 10 (sb = 0.415 µA) and the value of the slope of the calibration curve m = 1.24 µA/ppm. These values were replaced in LOD = 3s_b_/m. Therefore, the limit of detection (LOD) reached 1.0 ppm.

### 3.5. Consistency of the MIP/MWCNT Sensor

**Figure 8:**
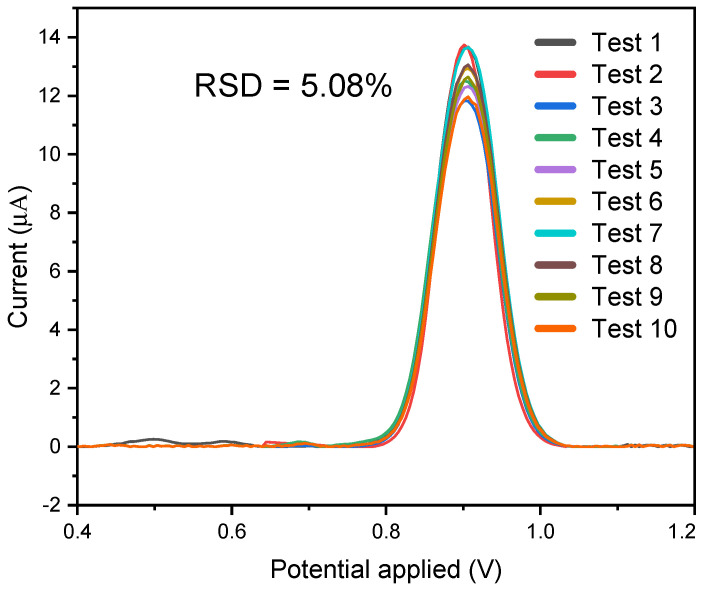
Repeatability graph obtained using the MIP/MWCNT sensor in 0.1 mol L^−1^ acetate buffer with acetonitrile (pH 5.0) containing 12 ppm carbendazim.

The consistency of the MIP sensor was assessed using optimized material created from the MIP mixture of the carbon nanotube in the presence of a 12 ppm carbendazim solution. The sensor’s consistency was assessed through a 10-cycle analysis, as shown in Figure 8 and below, and the measurements are repeatable while the relative standard deviation is 5.08%. Overall, the results reflected the analytical measurement and repeatability of raw data, and therefore, the modified sensor can also be used again and again.

### 3.6. Selectivity

**Figure 9:**
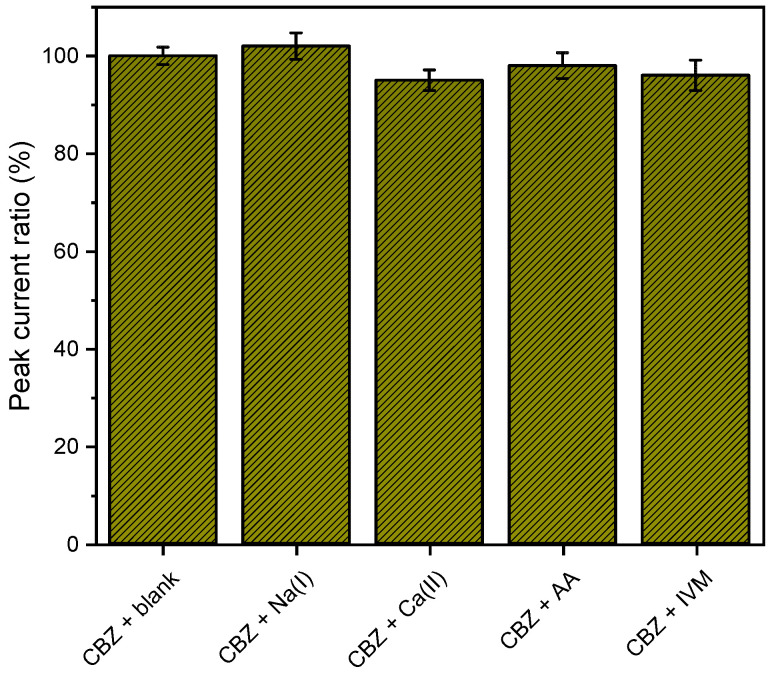
Selectivity graph obtained using the MIP/MWCNT sensor in 0.1 mol L^−1^ acetate buffer with acetonitrile (pH 5.0) containing 5 ppm of carbendazim (replicated *n* = 3).

The MIP/MWCNT sensor’s selectivity was evaluated in the presence of other interferents, including Na, Ca, L-ascorbic acid (AA), and ivermectin (IVM) (Figure 9), alongside carbendazim in a molar ratio of 1:1 (for each carbendazim concentration of 5 ppm). The outcomes are depicted in Figure 9. Notably, the constructed electrochemical (MIP/MWCNT) sensor exhibited superior selectivity compared to other compounds.

### 3.7. Application in Real Samples

The suggested polymer materials were evaluated by analyzing CBZ pesticide concentrations in river water and industrial wastewater. Two different concentrations of the spike were added to the various samples, and the HPLC technique was used to investigate them and compare the result with the electrochemical method. The recoveries found in the range of 97.34–101.35% are present in (Table 1) for HPLC, while for the electrochemical method, it is 94.28 to 103.72%. With regard to these results, the matrices did not produce any appreciable interference. The proposed polymer materials hold potential as an analytical tool for quantifying CBZ concentrations in real samples.

## 4. Conclusions

The incorporation of a molecularly imprinted polymer (MIP) on a glassy carbon electrode holds promise in the field of electroanalytical research for the development of biomimetic sensors. Its efficiency is further enhanced by multi-walled carbon nanotubes (MWCNTs), resulting in a low detection limit as low as 1.0 ppm. Remarkably, the results show a reproducibility of 5.08% (in terms of relative standard deviation) when employing differential pulse voltammetry (DPV) for *n* = 10. The proposed polymer materials were evaluated by analyzing the concentrations of the CBZ pesticide in both river water and industrial wastewater. The recoveries obtained, ranging from 94.28% to 103.72%, indicated a high level of stability. The findings of the present study reveal that the proposed biomimetic sensor is a highly efficient alternative tool well-suited for the simultaneous detection of CBZ in river water. The most striking advantages of the MIP sensing technique include its simplicity, rapid analysis, reproducibility, and selectivity for detecting the analyte.

## Figures and Tables

**Table 1:** Recovery values obtained for the analysis of wastewater samples using the HPLC method.

Sample	CBZ Concentration Added (ppm)	Recovery HPLC (%)	Recovery Electrochemical (%)
Industrial water	2.4	97.34	101.99
Industrial water	4.8	95.89	98.77
River Water	4.8	101.29	94.28
River water	2.4	101.35	103.72

## Data Availability

The data is contained within this communication.
